# A functional genomics screen of human B-cell differentiation reveals convergent mechanisms of inherited childhood leukemia predisposition

**DOI:** 10.64898/2026.08.06.743305

**Published:** 2026-08-07

**Authors:** Lara Wahlster, Anna-Lena Neehus, Andrew J. Lee, Soumyaa Mazumder, Pardiss Mehrzad, Susan Black, Luana Messa, Tanxin Liu, Charley Wang, Chen Weng, Alexis Caulier, Jensen Pak, Travis Fleming, Mateusz Antoszewski, Allison Zhang, Samuel A. Ha, Carmen Oleaga-Quintas, Adam J. de Smith, Vijay G. Sankaran

## Abstract

B-cell acute lymphoblastic leukemia (B-ALL) is the most common childhood cancer, yet the mechanisms by which inherited risk variants predispose to leukemia development remain poorly understood. A major challenge to studying these mechanisms has been the lack of model systems that faithfully capture the transient developmental states in which predisposition alleles are thought to act. Here, we establish a human B-cell differentiation platform from hematopoietic stem/progenitor cells that enables CRISPR-based engineering, recapitulates early B-cell lymphopoiesis, and enriches for rare developmental intermediates. By applying systematic perturbations with multiplexed single-cell transcriptomic profiling to mimic the effects of mutations in nine familial B-ALL predisposition genes, we decipher mechanisms by which B-cell development can be altered by such inherited variation to predispose to B-ALL. Through these studies, we identify convergent delays in B-cell differentiation at progenitor stages characterized by high-level RAG1/2 recombination activity. We propose that these delays at progenitor stages increase the likelihood that cells can undergo illegitimate RAG-mediated recombination to promote transformation, a finding consistent with similar rates of illegitimate RAG-associated genomic alterations in those with B-ALL associated with familial predisposition variants compared to sporadic cases.

## Full Text Availability

The license terms selected by the author(s) for this preprint version do not permit archiving in PMC. The full text is available from the preprint server.

